# Promoter and histone methylation and p16^INK4A^ gene expression in colon cancer

**DOI:** 10.3892/etm.2012.683

**Published:** 2012-08-24

**Authors:** EBRU ESIN YORUKER, UFUK MERT, DURSUN BUGRA, SUMER YAMANER, NEJAT DALAY

**Affiliations:** 1 Department of Basic Oncology, Oncology Institute and; 2 Department of Surgery, Istanbul Faculty of Medicine, Istanbul University, 34093 Capa, Istanbul, Turkey

**Keywords:** colon cancer, epigenetics, gene expression

## Abstract

The inactivation of the cyclin-dependent kinase inhibitor p16^INK4A^ gene by hypermethylation is observed in numerous types of cancer. New findings indicate that DNA and histone methylation act in concert in gene silencing. In this study, we investigated the methylation status of the p16^INK4A^ gene promoter and the histone 3 lysine 9 residue in the tumors and matched normal tissue samples from patients with colorectal cancer and analyzed their association with gene expression. The methylation and expression of the p16^INK4A^ gene were analyzed by real-time PCR, and histone methylation was analyzed by chromatin immunoprecipitation followed by real-time PCR. p16^INK4A^ expression was significantly higher in the tumors compared to normal tissue. Mono-, di- and trimethylation levels of the H3K9 residue were similar in the tumor and normal tissue samples. We did not observe any significant correlation between p16^INK4A^ methylation or expression and clinical parameters. Our results suggest that epigenetic modifications of the p16^INK4A^ gene and histone lysine methylation do not play a major role in colon carcinogenesis.

## Introduction

Genetic and epigenetic alterations underlie the pathogenesis of cancer. The disruption of epigenetic mechanisms leads to abnormal development and is involved in malignant transformation (1). Epigenetic changes include DNA methylation and histone modifications, which may affect gene expression by changing chromatin structure or modifying the DNA without altering the native nucleotide sequence. Since epigenetic changes are potentially reversible, they make attractive targets for therapeutic intervention (2).

Cytosine methylation usually only occurs in the CG dinucleotides which are unevenly distrubuted in the genome. The vast majority are located in the repetitive elements and heterochromatin. CpG islands in the promoter have a frequency of approximately five times greater than the rest of the genome (3). The CpG islands of transcriptionally active genes in normal cells are unmethylated. Methylation of this region leads to the modification of chromatin and transcriptional silencing of the gene.

Chromatin structure plays an integral role in the control of gene expression. The basic repeating unit of chromatin is the nucleosome which functions as a DNA packaging unit and transcripitional regulator (4). The amino terminal tails of the histones protrude out of the nucleosome and are subject to a variety of chemical modifications, including phosphorylation, acetylation, ubiquitination and methylation. These modifications affect the access of regulatory factors and complexes to chromatin and influence gene expression. Various sites of lysine methylation on the histones play essential roles in regulating chromatin structure and gene transcription. Methylation at lysine on histone H3 has recently been shown to be a marker of heterochromatin in all organisms (5). The methylation of H3K4, H3R17 and H4R3 has also been associated with the activation of transcription. By contrast, methylation of H3K9 has been correlated with gene silencing (6–9). H3K9 methylation is recognized by heterochromatin-associated proteins and is required to maintain the heterochromatic state. Degrees of methylation are differentially regulated and exert various functional outcomes. Studies have shown that modifications of histone H3 also contribute to gene silencing by switching between acetylation and methylation of the lysine 9 residue (10).

Epigenetic mechanisms play an important role in colon cancer and aberrant methylation of the p16^INK4A^ gene is commonly observed (11,12). The p16^INK4A^ gene [also known as cyclin-dependent kinase inhibitor 2A (*CDKN2A*)] is a member of the INK4A/ARF family of suppressors of cyclin-dependent kinases (CDKs) which plays an important role in the G1-S transition by binding to CDK4 and CDK6 and inhibiting the progression of the cell cycle (13,14). Inactivation of the p16^INK4A^ gene is considered to be the second most common defect in human cancer (15,16). The p16^INK4A^ gene is inactivated by homozygous deletions, point mutations and preferentially by methylation of the gene promoter (17,18). p16^INK4A^ promoter methylation and subsequent gene silencing have been reported in various cancer types (19–23). The differences between the methylation states of the histone H3 lysine 9 have been associated with repression (24) and activation (25) of important genes. In this study we aimed to investigate the expression of the p16^INK4A^ gene and the methylation status of the promoter region and the histone H3 lysine 9 residue. To our knowledge this is the first study in the literature to analyze these parameters in matched tumor samples.

## Materials and methods

In this study tumor specimens from 71 patients with colorectal cancer (mean age, 61.04±14.8) and matched normal tissue samples were analyzed. The characteristics of the patients are summarized in Table I. The study was approved by the Istanbul Faculty of Medicine Ethics Committee (2006-504).

### Sodium bisulfite modification of DNA

Genomic DNA was extracted from the tissue samples. The quality of DNA was evaluated spectrophotometrically at 260/280 nm. The bisulfite conversion was performed according to the manufacturer’s instructions using the Methylamp™ DNA Modification kit (Epigentek, Brooklyn, NY, USA). During this process all unmethylated cytosines are deaminated, sulfonated and converted to uracil, while 5-methylcytosines remain unaltered. The bisulfite-modified DNA was stored at −20°C until use. DNA from placenta treated with *Sss*I methyltransferase (New England Biolabs, Ipswich, MA, USA) was used as a standard for the methylated reaction.

### Methylation-specific PCR

To increase the sensitivity of methylation detection, we used a 2-step methylation-specific PCR approach, in which the outside primer pairs selectively amplify bisufite-modified DNA, irrespective of the methylation status. A second reaction then uses specially designed nested primer pairs to discriminate between the methylated and unmethylated alleles. The sequences of the outside primers were: forward; 5′-GGAGGAAGAAAGAGGAGGGGT-3 and reverse; 5′-CTACCTAATTCCAATTCCCCT-3′ (Integrated DNA Technologies, Coralville, IA, USA). Amplification was performed on a thermal cycler (Techne, Inc., Burlington, NJ, USA) as follows: denaturation for 5 min at 94°C and 35 cycles of denaturation at 94°C for 30 sec, annealing at 64°C for 30 sec and extension at 72°C for 30 sec. PCR reactions were carried out in a total volume of 25 μl containing 1X reaction buffer, 2 mM MgCl_2_, 200 μM deoxynucleotide triphosphate, 400 pmol of each primer, 1.5 unit Taq polymerase (Fermentas, Vilnius, Lithuania) and 3 μl modified DNA.

The second round PCR reaction (quantitative step) was performed using real-time PCR (LightCycler^®^; Roche, Mannheim, Germany). The PCR mix contained 1 μl of PCR product from the first step, 1X hybridization probes, 2 mM MgCl_2_, 0.5 μM of each primer and 0.2 μM of each probe in a total volume of 15 μl. Following denaturation at 95°C for 10 min, amplification was performed by 45 cycles of denaturation at 94°C for 10 sec, annealing at 50°C for 10 sec and extension at 72°C for 10 sec. Standard curves were constructed using serial dilutions of methylated placental DNA. The sequences of the primers were: forward; 5′-TAGAGGGTGGGGCGGATCGCG-3′ and reverse; 5′-CCAAAATCGCCCGCCATCCC-3′. The fragment of the p16^INK4A^ gene amplified by the second primer pair covers 12 CpG dinucleotides. The sequences of probes were 5′-CCGCCGCCCGCTACCTACTCT-FL, 5′-LC640-CCCTCTCCGCAACCGCCGAAC-PH. The two probes were designed to hybridize to the same strand between two unlabeled primers. Fluorescence monitoring of amplification is based on the concept that a signal is generated when fluorescence resonance energy transfer occurs between two adjacent fluorescently-labeled sequence-specific hybridization probes. The emitted fluorescence is measured and is proportional to the quantity of specific target sequences in the reaction mixture. The methylation levels were calculated by comparing the data from the samples with the standards using the LightCycler Relative Quantification Software (Roche).

### Chromatin immunoprecipitation (ChIP)

For the ChIP assay the method available on the UC Davis Genome Center web site (26) was used with certain modifications. Briefly, tissue samples were treated with 1% formaldehyde for 15 min to cross-link histones to DNA. After washing, the pellets were resuspended in the lysis buffer [5 mM PIPES (pH 8.0), 85 mM KCl, 0.5% NP-40, protease inhibitors (aprotinin, leupeptin, PMSF)] and nuclei lysis buffer [50 mM Tris-Cl (pH 8.1), 10 mM EDTA, 1% SDS, protease inhibitors], respectively. Each sample was sonicated 7 times for 8 sec each. The lysate was then divided into 3 fractions; which were incubated overnight at 4°C with 2 μg of the appropriate antibodies for H3K9 mono-, di- and trimethylation (Millipore, Billerica, MA, USA), respectively. The pellets were washed with dialysis buffer [2 mM EDTA, 50 mM Tris-Cl (pH 8.0)] and IP wash buffer [100 mM Tris-Cl (pH 9.0), 500 mM LiCl, 1% NP-40, 1% deoxycholic acid] and were resuspended in the elution buffer (50 mM NaHCO_3_, 1% SDS). DNA was extracted using the phenol-chloroform method, ethanol precipitated and resuspended in 20 μl of water. The PCR amplification of DNA was performed using the LightCycler 1.2 (Roche) and the double-stranded DNA binding dye SYBR-Green I as the fluorescent molecule. The PCR mix contained 1.2 μl of SYBR Green mix, 2 mM MgCl_2_, 0.5 μM each primer and 4 μl DNA in a total volume of 12 μl. Amplification was performed by denaturation at 95°C for 10 min followed by 45 cycles of denaturation at 94°C for 10 sec, annealing at 60°C for 10 sec and extension at 72°C for 10 sec. The sequences of the primers were: forward; 5′-AGACAGCCGTTTTACACGCAG-3′ and reverse; 5′-CACCGAGAAATCGAAATCACC-3′. Known concentrations of serial diluted DNA were used for the standard curve. To increase the specificity of SYBR Green I detection, we performed a melting curve analysis of the amplification reaction. The area below each of the peaks representing the relative amount of nucleic acids was calculated using the LightCycler Data Analysis Software (Roche).

### Analysis of p16^INK4A^ gene expression

RNA samples were available from tumors of 41 patients. p16^INK4A^ mRNA levels were measured by real-time quantitative RT-PCR using the LightCycler 480 instrument (Roche Applied Science, Indianapolis, IN, USA) and UPL probes.

Total RNA was extracted from the tumor and normal tissue samples using the SV Total RNA Isolation system (Promega Corporation, Madison, WI, USA) as per the manufacturer’s instructions. RNA from each sample was used to generate cDNA using the RevertAid First Strand cDNA Synthesis kit (Fermentas). Briefly, 1 μg RNA and 1 μl of oligo(dT) were used as starting materials, heated and kept at 70°C for 5 min. The samples were then chilled on ice, the other components (5X reaction buffer, ribonuclease inhibitor and dNTP mix) were added and the samples were incubated at 37°C for 5 min. Then 1 μl reverse transcriptase (200 U/μl) was added and the samples were incubated at 42°C for 60 min. The reaction was inactivated at 70°C for 10 min.

The probes were designed using the Lightcycler Probe Design Software 2.0 (Roche). The HPRT gene was used as the internal control. The PCR mix of 20 μl was prepared by adding to 5 μl cDNA template, 2.5 μM of each of the forward and reverse primers (final concentration) and 10 μl of the probe master mix. The sequences of the forward and reverse primers were: 5′-GTGGACCTGGCTGAGGAG-3′ and 5′-CTTTCAATCGGGGATGTCTG-3′. The PCR conditions were: one cycle at 95°C for 10 min, 45 cycles of denaturation at 95°C for 10 sec, annealing at 60°C for 30 sec and elongation at 72°C for 1 sec with subsequent cooling at 40°C for 30 sec. The results were analyzed by basic relative quantification.

The tumor and normal samples were compared using the Mann-Whitney U test; associations with clinical parameters were evaluated using the Kruskal-Wallis test and Spearman’s rank correlation.

## Results

**Figure 1:**
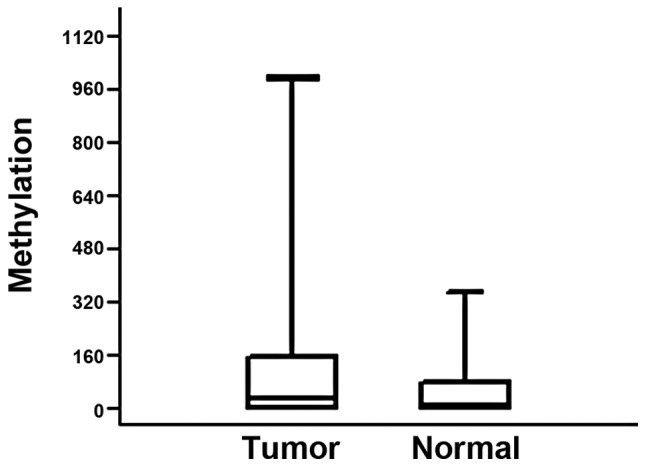
Methylation of the p16^INK4A^ gene promoter in the tumor and normal tissue samples shown in fluorescence units. The boxes indicate the upper and lower quartiles.

Tissue samples were collected from 71 patients with colorectal cancer who underwent surgery for tumor resection. Methylation of the p16^INK4A^ gene promoter was analyzed by quantitative real-time PCR in the tumors and matched normal tissue samples from the patients. Methylation at the p16^INK4A^ promoter was higher in the tumors from 38 patients (53.5%) when compared with the normal tissue of the same individuals (Fig. 1). The median methylation levels were 24 for tumor tissue and 3.5 for normal tissue. However, due to the wide range of the values the difference was not significant (P=0.06). We also did not identify any significant correlation between the methylation levels and clinical parameters, including lymph node status, metastasis, stage and tumor location.

**Figure 2:**
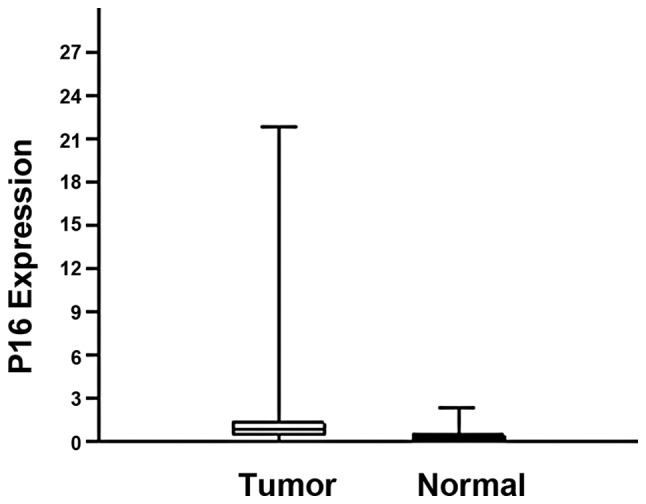
Expression of the p16^INK4^ gene in the tumors and normal tissue. The vertical axis indicates the normalized fluorescence signal. The boxes indicate the upper and lower quartiles.

The expression of the p16^INK4A^ gene was investigated in 41 samples by real-time PCR. p16^INK4A^ expression was higher in the tumors than normal tissue in 31 patients (75.6%). The median expression levels were 0.869 and 0.312 for the tumor and normal tissue samples, respectively. The difference was significant (P=0.0005; Fig. 2). No association was observed between the methylation status and expression of the p16^INK4A^ gene both for the tumor (P=0.315) and normal tissue (P=0.209) samples.

**Figure 3:**
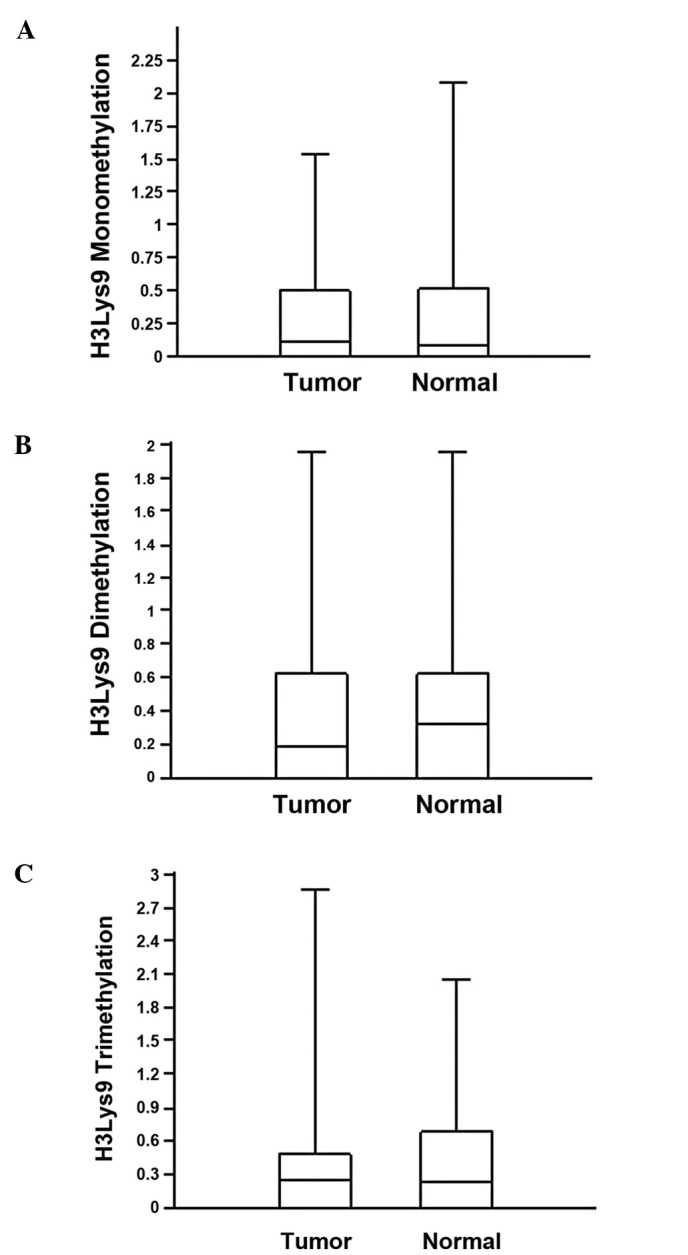
H3Lys9 methylation in the tumors and normal tissue. (A) Monomethylation, (B) dimethylation and (C) trimethylation. The vertical axis shows the area under the curve which represents of the amount of specific methylation.

To determine the correlation between p16^INK4A^ methylation and chromatin remodelling, mono-, di- and trimethylation of the H3K9 residue was analyzed by chromatin immunoprecipitation and real-time PCR. The values of H3K9 mono-, di- and trimethylation for the normal and tumor tissues were similar (Table II). The median values of monomethylation for the tumor and normal tissue samples were 0.112 and 0.08, respectively (Fig. 3A). Median values for di- and trimethylation in the tumors and normal tissue were 0.187 vs. 0.325 and 0.257 vs. 0.234, respectively (Fig. 3B and C). The difference between the methylation levels of H3K9 in the normal and tumor tissues was not significant (Mann-Whitney U test, P>0.05). The expression level of the p16^INK4A^ gene was not associated with mono-, di- or trimethylation for the tumor (P=0.404, P=0.457 and P=0.465, respectively) or normal (P=0.288, P=0.62 and P=0.667, respectively) samples.

## Discussion

Epigenetic mechanisms involving DNA methylation and histone modifications are closely associated but the critical initiating events in silencing remain undefined. Malignant cells are characterized by a localized increase in methylation in CpG island-associated promoters (27,28). Several lines of evidence indicate that alterations in histone modifications are crucial in cancer development and progression (29). In certain organisms all DNA methylation is dependent on H3K9 methylation (30). However, it remains unclear whether DNA methylation actually directs H3K9 methylation or whether it acts independently to silence gene expression in the absence of H3K9 methylation.

Methylation of the p16^INK4A^ gene as an epigenetic event has been reported in several tumor types (19–23,31). In this study methylation levels of the p16^INK4A^ gene promoter and of the histone H3K9 were analyzed in colorectal tumors and normal colon samples. To date, this is the first study to investigate DNA and histone methylation in a series of matched human tumors and normal tissue samples.

Expression of the p16^INK4A^ gene was significantly higher in tumor samples. This finding is consistent with the observation that promoter methylation levels were not different between the tumor and normal samples since methylation may also be detected in normal colon cells (32). The frequency of the hypermethylated tumors was 53% in this study. This value is in agreement with recent reports (33–36) but there are also studies reporting lower values in the literature (32,37,38). These differences may be explained by the high sensitivity of the methods used in our study. The use of primers and probes designed for the methylated sequences and application of nested PCR increases the specificity of the method markedly and makes it possible to detect low levels of methylated sequences (39). We also observed methylation in 45.3% of the normal tissue samples. A number of recent reports have confirmed that methylation is also observed in normal tissue (32,40,41). It has been suggested that methylation in normal tissue may be associated with increasing age (40,42). Suppression of p16^INK4A^ expression by methylation may be much lower or even absent in aging colon tissue (43) since increased p16^INK4A^ expression which accompanies promoter methylation has been reported in aging cells (44,45). This phenomenon is thought to play a role in the aging process by promoting senescence and the decline in gene function (46).

The epigenetic reprogramming of cancer cells occurs early in oncogenesis and is difficult to study in clinical samples. The correlation between DNA methylation and histone lysine methylation during the development of colon cancer remains unclear. There are numerous studies reporting findings in various fields. One of the first studies suggested a direct association between DNA methylation and histone lysine methylation in a cell line (47). In plants DNA methylation is sufficent for gene silencing (48). In prostate cancer cells, the effect of H3K27 trimethylation on gene silencing is independent of DNA methylation (49). It has also been demonstrated that silencing of p16^INK4A^ and H3K9 methylation precede DNA methylation (50). Histone H3K9 trimethylation is essential for the cells to survive radiation damage. Impairment of lysine 9 methylation may increase cancer risk by altering the efficiency of DNA repair (51). When we compared H3K9 methylation levels in the tumor and normal tissue samples no significant difference was observed. Analysis of H3K9 methylation indicated that various levels of methylation exist within the tissue. The rate of monomethylation in the tissue samples was lowest while the di- and trimethylation levels were similar. Conflicting results in the literature show that epigenetic mechanisms may also differ from organism to organism or from gene to gene. In the present study we could not detect any correlation between DNA methylation and H3K9 methylation. We also did not observe any correlation between the p16^INK4A^ promoter or H3K9 methylation and the clinical parameters. The lack of association with clinical characteristics is in accordance with recent reports (16,32).

In our study expression of the p16^INK4A^ gene was higher in the tumor tissue than in normal tissue in 75% of the patients. These data are in agreement with studies showing that p16^INK4A^ expression in colon carcinoma is frequently observed (31,52). It has even been reported that the majority of colorectal tumors express the p16^INK4A^ protein (53). Coexistence of p16^INK4A^ methylation and expression has also been demonstrated (54,55). Similar findings have been confirmed by various studies in which p16^INK4A^ expression in excess of 80% of the colon tumors has been observed (16,56,57). To the best of our knowledge, this is the first study comparing methylation and histone modifications of the p16^INK4A^ gene in colon tumors and corresponding normal tissue. Our results suggest that although epigenetic alterations of the p16^INK4A^ gene are frequent in colon tumors they appear to accompany other genetic alterations which play a causative role in colon carcinogenesis.

## Figures and Tables

**Table I:** Clinical characteristics of the patients.

Characteristics	No. of patients	%
Stage (n=46)		
I	3	6.5
II	5	10.8
III	16	34.7
IV	19	41.3
Tumor location (n=71)		
Colon	31	43.6
Rectum	30	42.2
Rectosigmoid	10	14
Lymph node status (n=46)		
Negative	11	23.9
Positive	35	76.08
Distant metastasis (n=46)		
Negative	27	58.6
Positive	19	41.3
Differentiation (n=36)		
High	5	13.8
Middle	28	77.7
Low	3	8.3

**Table II:** H3K9 methylation frequencies and median values in the tumors and normal samples.

	Monomethylation		Dimethylation		Trimethylation	
Sample	%	Median	%	Median	%	Median
Tumor	56.3	0.112	63.3	0.187	61.9	0.257
Normal	53.5	0.08	61.9	0.325	57.7	0.234
